# Supplementary material for: Electrocardiographic interpretation by emergency medical services professionals in Saudi Arabia: A cross sectional study
Source: PLoS One. 2023 Oct 19;18(10):e0292868. doi: 10.1371/journal.pone.0292868 (PMC10586609; doi:10.1371/journal.pone.0292868)
Supplement: S1 Checklist — (DOCX) [file pone.0292868.s001.docx]

STROBE Statement—checklist of items that should be included in reports of observational studies

|  | Item No. | Recommendation | Page  No. | Relevant text from manuscript |
| --- | --- | --- | --- | --- |
| **Title and abstract** | 1 | (*a*) Indicate the study’s design with a commonly used term in the title or the abstract | Page 1 (Lines 1-2) | Electrocardiographic interpretation by emergency medical services professionals in Saudi Arabia: A cross sectional study |
|  |  | (*b*) Provide in the abstract an informative and balanced summary of what was done and what was found | Pages 2-3 (Lines 37-65) | **Abstract**  **Background**  Management of acute myocardial infarction (AMI) and cardiac arrythmias in prehospital settings are largely determined by providers of emergency medical services (EMS) who can proficiently interpret the electrocardiography (ECG). The aim of this study was to assess the ECG competency of EMS providers in Saudi Arabia.  **Methods**  Between Aug and Sep 2022, we invited all EMS providers working for the Saudi Red Crescent Authority in Makkah, Riyadh, and Sharqiyah regions to complete a cross-sectional survey. The survey was used to assess the ability of EMS providers in interpreting 12 ECG strips. Characteristics and ECG competency were summarized using descriptive statistics. Differences in ECG competency across paramedics with lower and higher qualifications were assessed.  **Results**  During the study period, 231 participants completed the survey and all were included. Overall mean age was 33.4, and most participants were male (94.8%). Nearly half of the participants were paramedics with associate degree and 46.4% were paramedics with higher degrees. The average rate of the correct answers to the 12 ECG strips was 43.3% (95% CI: 35.4%, 51.3%). Atrial flutter, ventricular fibrillation, atrial fibrillation, 3^rd^ degree heart block, and ventricular tachycardia were identified by 52.8%, 60.2%, 42.0%, 40.7%, and 49.4% of the participants, respectively. The strip with an AMI was identified by 41.1% while a pathological Q wave and ventricular extrasystole was identified by 19.1% and 24.7%, respectively. Paramedics with higher qualifications were as 28.0%-61.0% as likely to correctly interpret the 12 ECG strips compared to those with associate degree (p-value across all variables was ≤ 0.001).  **Conclusion**  While the majority of participants in our region were unable to correctly identify the 12 ECG strips, paramedics with higher qualifications were. Our findings indicate that there is a need for a structured-ECG training on a regular basis for our EMS providers. |
| Introduction | | | |  |
| Background/rationale | 2 | Explain the scientific background and rationale for the investigation being reported | Pages 4-5 (Lines 69-93) | **Background**  Ischemic heart disease (IHD) accounted for 9.4 million deaths in 2021, worldwide.^1^ Early screening and prevention interventions for individuals at high risk of IHD in the primary healthcare settings do not reduce mortality.^2^ While many people normally live and cope with a heart disease, the heart disease can progress to life-threatening conditions.^3^ Acute myocardial infarction (AMI) and cardiac arrythmias are examples of these and often seen by emergency medical services (EMS) personnel in prehospital settings. Using the 12-lead electrocardiography (ECG), EMS personnel can improve survival and quality of life outcomes of patients following AMI and cardiac arrythmias.^4, 5^ However, not every prehospital care provider is competent in interpreting the ECG.^6-9^  A number of studies have been carried out in US, Canada, and Australia to assess the competency of EMS personnel in diagnosing patients with AMI using the 12-lead ECG.^10^ In Boston, US, paramedics with basic and advanced life support skills were able to identify 80% of patients with confirmed AMI.^8^ Similarly, primary care paramedics were able to accurately diagnose patients with AMI in 79% of the cases in Simcoe County, Canada.^7^ Additionally, mobile intensive care paramedics from Ambulance Victoria, Australia diagnosed 58% of AMI cases.^9^ Although prehospital care providers from these regions have not correctly detected 20%-40% of the cases, such estimates were derived from studies published before 2013. They were also pooled from studies conducted across regions with developed EMS systems. As such, it is unclear whether paramedics in a developing EMS system such as the EMS of the Saudi Red Crescent Authority (SRCA) would reach better or similar figures. In addition, since a large proportion of prehospital care providers in the SRCA are either paramedics with associate or advanced degrees, it is not known if the level of education would have an impact on the ECG interpretation skills. |
| Objectives | 3 | State specific objectives, including any prespecified hypotheses | Page 5 (Lines 94-97 | This study aimed to assess the ability of prehospital care providers in the SRCA to correctly interpret ECG strips using a previously published ECG competency survey.^11^ Differences in ECG competency across levels of paramedic education will also be examined. |
| Methods | | | |  |
| Study design | 4 | Present key elements of study design early in the paper | Page 6 (Lines 99-113) | *Study design*  This was a cross-sectional study design. All EMS personnel working for the SRCA in Makkah, Riyadh, and Sharqiyah regions were invited through their SRCA email account to voluntarily complete an electronic survey between 27^th^ August 2022 and 21^st^ September 2022. Each EMS provider included in this study has formally consented to participate at the start of completing the survey. Information for each participant was obtained and maintained in a de-identified data format. Participants from other regions were excluded. This study has ethics approval from the Institutional Review Board of SRCA (No: 22-66E).  Since the number of our population is a continuous variable, we decided to use the Adam’s 2020 formula for calculating the sample size.^12^ Across the three regions, there were ≤1500 EMS personnel. At a value of 0.03 for the degree of accuracy (e), 4.0 standard deviations (σ), 1.96 for the t-value with a two-tailed 95% confidence intervals (t), and 0.06 for the adjusted margin of error (ԑ), our sample size (n) should include ≈ 227 participants. Where ԑ = (σ(e)/t) and n = 1500/1+1500(ԑ)^2^. |
| Setting | 5 | Describe the setting, locations, and relevant dates, including periods of recruitment, exposure, follow-up, and data collection | Pages 6-7 (Lines 114-137) | *Settings*  The SRAC is the national primary provider of EMS across Saudi Arabia. Prehospital care ambulance units manned with basic and advanced life support personnel respond to emergency cases. Personnel of EMS operate under the clinical practice guidelines of the SRCA ([www.srca.org.sa](http://www.srca.org.sa)). All patients with chest pain or epigastric discomfort or presenting with signs and symptoms of acute coronary syndrome must be assessed before leaving the scene using the 12-lead ECG. The ECG strips are then transmitted through an electronic communication platform to the online medical director for further medical consultations.  Responding to cases with cardiovascular disease and acute coronary syndrome by EMS personnel is within the National EMS Scope of Practice ([www.srca.org.sa](http://www.srca.org.sa)). All EMS personnel working for the SRCA are registered with the Saudi Health Council for Health Specialties (SHCFHS).^13^  *Survey*  The 12-item questionnaire developed by Coll-Bandell et al. in 2017 was administered to our study population with minor modifications. Permission to use the survey was acquired from the primary author. The survey was originally used to assess the ability of emergency nurses to interpret the 12 ECG strips. The survey is comprised of two sections. Section one was designed to collect demographical data such as age, sex, location, years of experience, and previous ECG training course. Section two was designed to assess the ECG knowledge and interpretation skills of the participants. In this section, participants were asked to answer two general questions about the waveform shape of the ECG and interpret 10 ECG strips with different levels of complexity. One correct answer out of four possible answers was developed for each question. |
| Participants | 6 | (*a*) *Cohort study*—Give the eligibility criteria, and the sources and methods of selection of participants. Describe methods of follow-up  *Case-control study*—Give the eligibility criteria, and the sources and methods of case ascertainment and control selection. Give the rationale for the choice of cases and controls  *Cross-sectional study*—Give the eligibility criteria, and the sources and methods of selection of participants | Page 6 (Lines 99-113) | As described in the study design, item number 4. |
|  |  | (*b*) *Cohort study*—For matched studies, give matching criteria and number of exposed and unexposed  *Case-control study*—For matched studies, give matching criteria and the number of controls per case | NA |  |
| Variables | 7 | Clearly define all outcomes, exposures, predictors, potential confounders, and effect modifiers. Give diagnostic criteria, if applicable | NA | The study is descriptive in nature. |
| Data sources/ measurement | 8* | For each variable of interest, give sources of data and details of methods of assessment (measurement). Describe comparability of assessment methods if there is more than one group | Page 7-8 (Lines 127-150) | We have provided all the information necessary to describe variables and how are they going to be assessed (Item number 5). |
| Bias | 9 | Describe any efforts to address potential sources of bias | NA |  |
| Study size | 10 | Explain how the study size was arrived at | Page 6 (Lines 108-113) | Since the number of our population is a continuous variable, we decided to use the Adam’s 2020 formula for calculating the sample size.^12^ Across the three regions, there were ≤1500 EMS personnel. At a value of 0.03 for the degree of accuracy (e), 4.0 standard deviations (σ), 1.96 for the t-value with a two-tailed 95% confidence intervals (t), and 0.06 for the adjusted margin of error (ԑ), our sample size (n) should include ≈ 227 participants. Where ԑ = (σ(e)/t) and n = 1500/1+1500(ԑ)^2^. |

Continued on next page

| Quantitative variables | 11 | Explain how quantitative variables were handled in the analyses. If applicable, describe which groupings were chosen and why | Page 7-8 (Lines 138-150) | *Statistical analysis*  Characteristics and ECG knowledge and interpretation skills of the study population were summarized using descriptive statistics. Continuous variables were reported as means and standard deviations. Categorical variables were reported as counts and percentages. We stratified our population into two groups to assess differences in ECG interpretation skills across stratifications. Paramedics with associate degree were compared to paramedics with bachelor’s and master’s using the Chi-square test (X^2^). Since previous ECG training course and ECG mode of instruction may produce some confounding effects on our assessment, we performed a sensitivity analysis. We assessed whether such variables would significantly differ between the two groups using the X^2^ test. A two-tailed p-value of < 0.05 was considered statistically significant. All statistical analyses were carried out using STATA statistical software, version 16.0 (Statacorp, College Station, Texas, USA). |
| --- | --- | --- | --- | --- |
| Statistical methods | 12 | (*a*) Describe all statistical methods, including those used to control for confounding | Page 7-8 (Lines 138-150) | As described above. |
|  |  | (*b*) Describe any methods used to examine subgroups and interactions | Page 7-8 (Lines 138-150) | As described in item number 11. |
|  |  | (*c*) Explain how missing data were addressed | NA | There is no missing data. |
|  |  | (*d*) *Cohort study*—If applicable, explain how loss to follow-up was addressed  *Case-control study*—If applicable, explain how matching of cases and controls was addressed  *Cross-sectional study*—If applicable, describe analytical methods taking account of sampling strategy | NA |  |
|  |  | (*e*) Describe any sensitivity analyses | Page 7-8 (Lines 138-150) | As described in item number 11. |
| Results | | | | |
| Participants | 13* | (a) Report numbers of individuals at each stage of study—eg numbers potentially eligible, examined for eligibility, confirmed eligible, included in the study, completing follow-up, and analysed | Page 9 (Lines 152-153) | Between 27^th^ Aug 2022 and 21^st^ Sep 2022, 231 EMS personnel completed the survey, and all were included in the final analyses. |
|  |  | (b) Give reasons for non-participation at each stage | NA |  |
|  |  | (c) Consider use of a flow diagram | NA |  |
| Descriptive data | 14* | (a) Give characteristics of study participants (eg demographic, clinical, social) and information on exposures and potential confounders | Page 9-13 (lines 154-185) | **Baseline characteristics**  Table 1 presents baseline characteristics of the study population. The overall mean age was 33.4 and most participants were male (94.8%). The majority of respondents were from Riyadh region (60.6%) and 34.6% had 1-5 years of work experience. Paramedics with associate degree nearly represented half of the study population (48.1%) wile paramedics with undergraduate and graduate degree represented the other half (46.4%). About 60.0% of the participants reported that they have undertaken ECG training course. Of those, 44.9% reported that the ECG training course they have taken was ≤ 1 year ago and 68.4% reported that the ECG mode of instruction was face-to-face. The majority of the participants received less than 10 hours of ECG training (69.8%).  **Interpretation of the ECG**  The ability of the EMS providers to interpret the ECG is presented in Table 2. Approximately, sixty percent of the participants were able to list the correct order of the waveform shape on the ECG and 52.4% were able to assess the value of P-wave presentation on the strip. Atrial flutter, ventricular fibrillation, atrial fibrillation, 3^rd^ degree heart block, and ventricular tachycardia were identified by 52.8%, 60.2%, 42.0%, 40.7%, and 49.4% of the study population, respectively. Forty-one percent of the participants were able to identify the patient with acute myocardial infarction and < 25.0% were able to identify patients with pathological Q wave and ventricular extrasystole. On average, the rate of participants who were able to correctly identify the ECG strips was 43.3% (95% CI: 35.4%, 51.3%).  **Differences of ECG interpretation between EMS groups**  Table 3 compared personnel of EMS with associate degree to those with bachelor’s and master’s. Across the 12-item questionnaire, paramedics with higher qualifications were as 28.0%-61.0% as likely to correctly answer the questions and interpret the ECG strips compared to those with associate degree (p-value across all variables was ≤ 0.001). Differences in previous ECG training course and ECG mode of instruction between the two groups were not statistically significant (Table 4). |
|  |  | (b) Indicate number of participants with missing data for each variable of interest | NA |  |
|  |  | (c) *Cohort study*—Summarise follow-up time (eg, average and total amount) | NA |  |
| Outcome data | 15* | *Cohort study*—Report numbers of outcome events or summary measures over time | NA |  |
|  |  | *Case-control study—*Report numbers in each exposure category, or summary measures of exposure | NA |  |
|  |  | *Cross-sectional study—*Report numbers of outcome events or summary measures | Page 9-13 (lines 154-185) | As described in item number 14 |
| Main results | 16 | (*a*) Give unadjusted estimates and, if applicable, confounder-adjusted estimates and their precision (eg, 95% confidence interval). Make clear which confounders were adjusted for and why they were included | NA |  |
|  |  | (*b*) Report category boundaries when continuous variables were categorized | NA |  |
|  |  | (*c*) If relevant, consider translating estimates of relative risk into absolute risk for a meaningful time period | NA |  |

Continued on next page

| Other analyses | 17 | Report other analyses done—eg analyses of subgroups and interactions, and sensitivity analyses | Page 9-13 (lines 154-185) | As described in item number 14 |
| --- | --- | --- | --- | --- |
| Discussion | | | | |
| Key results | 18 | Summarise key results with reference to study objectives | Page 15 (192-198) | In this cross-sectional study, the average rate of the correct answers to the 12 ECG strips for all participants (n=231) was 43.3% (95% CI: 35.4%, 51.3%). Of the 12 ECG strips, more than 74.0% of the study population were not able to correctly identify cardiac rhythms with pathological Q wave and ventricular extrasystole. Paramedics with bachelor’s and master’s degree (n=107), on overage, had a rate of correct answers at 65.4% (95% CI: 54.1%, 76.8%) while it was at 25.2% (95% CI: 18.3%, 32.1%) for paramedics with associate degree (n=111). |
| Limitations | 19 | Discuss limitations of the study, taking into account sources of potential bias or imprecision. Discuss both direction and magnitude of any potential bias | Page 18 (Lines 244-257). | **Limitations**  This study has some potential limitations. First, our data were obtained from regions that are highly populated with a high demand for EMS. Some EMS stations across these regions receive more calls compared to other stations. It is therefore possible that some of our participants were less or more exposed to cardiac cases and arrythmias. Exposure to cardiac cases may have produced some confounding effect that could underestimate or overestimate our rates. Second, prehospital care providers in our region generally receive formal education in cardiology and are trained to interpret the ECG, but the same level of teaching and training for all participants cannot be confirmed. Our participants may be of low, medium, or high level of literacy in ECG or a mixture of all. As such, although our finding indicates that paramedic with higher degree were better than those with associate degree, we are not completely certain. Third, the survey was emailed to participants to independently self-complete the survey. It is therefore difficult to conclude that no participant received any assistance at completing the survey. |
| Interpretation | 20 | Give a cautious overall interpretation of results considering objectives, limitations, multiplicity of analyses, results from similar studies, and other relevant evidence | Pages 15-17 (Lines 199-242) | Our participants had an overall lower rate of correct answers (43.3%) compared to the rate reported in a study with similar study design. Authors of the original survey tool used in our study have reported a rate of correct answers at 86.8%.^11^ In that study, all participants were undergraduate nurses working in emergency department. It is common for many healthcare professionals with undergraduate degree to receive some formal education in cardiology and ECG interpretation. It is also possible for nurses to have gained more experience in ECG interpretation through their rich exposure to cardiac cases that are often seen in the emergency rooms or hospital settings. However, our finding seems comparable to a study from Sweden. In that study, 54.0% of ambulance nurses have successfully provided the correct answers to the 16 ECG strips they have been provided with.^14^ Although, most of our study population were paramedics with different levels of education, our participants and participants from the Swedish study received no formal ECG training right before taking the test and were not often much exposed to cardiac cases.  Prehospital care providers in our region are more likely to misinterpret the ECG rhythms with premature ventricular contraction and patterns indicating a wide range of differential diagnosis. Our findings are similar to that reported in other regions from Saudi Arabia. Across 427 medical students from Umm Al-Qura university, Saudi Arabia, the ventricular extrasystole and pathological Q wave were not identified by nearly 75.1% of the participants.^15^ Medical interns were also not able to correctly diagnose patients with such rhythms (75.7%) in a study carried out across 14 medical universities in Saudi Arabia.^16^ Conversely, our findings differ from that reported in Iran region. In that report, 55.7% were not able to identify ECG strips with pathological Q wave and ventricular extrasystole.^17^ However, 74.0% of the included population were nurses and physicians. Hospital-based working healthcare professionals are likely to be more trained and experienced in ECG interpretation compared to those who are not.^18^ While this could partly explain the deficiency of ECG interpretation across our population, there are also some concerns that ECG training curriculum is often not standardized and tailored to a specific profession with a range of mastery levels.^18, 19^ It is possible that our participants were not up to the advanced levels of ECG interpretation.  Paramedics with higher levels of education were 40.2% more proficient in interpreting the ECG than those with associate degree. This is often common across many healthcare disciplines. For example, paramedic students at year four from King Saud University, Saudi Arabia, were 23.0% more competent in ECG than those at year two.^20^ In medicine discipline, the accuracy of ECG interpretation by cardiologists was 32.9%, 19.1% and 6.4% greater than medical students, residents, and practicing physicians, respectively.^18^ In the nursing filed, the level of knowledge in ECG interpretation was 27.3% higher in a group of nurses with an undergraduate degree relative to nurses with diploma.^21^ Our finding suggests that higher education is an important factor for paramedics’ performance when dealing with cardiac patients. Undergraduate and graduate education for prehospital care providers, paramedics, is the norm in many regions such as UK, Australia. New-Zealand and Saudi Arabia.^13, 22^ There is also some evidence from the National EMS Certification, US, indicating that paramedic education is shifting from associate degree to bachelor’s and postgraduate between 2017 and 2019.^23^ |
| Generalisability | 21 | Discuss the generalisability (external validity) of the study results | Page 18 (Lines 244-257). | As described in item number 19. |
| Other information | |  | | |
| Funding | 22 | Give the source of funding and the role of the funders for the present study and, if applicable, for the original study on which the present article is based | Page 19 (Lines 272-273) | **Funding source**  None to declare. |

*Give information separately for cases and controls in case-control studies and, if applicable, for exposed and unexposed groups in cohort and cross-sectional studies.

**Note:** An Explanation and Elaboration article discusses each checklist item and gives methodological background and published examples of transparent reporting. The STROBE checklist is best used in conjunction with this article (freely available on the Web sites of PLoS Medicine at http://www.plosmedicine.org/, Annals of Internal Medicine at http://www.annals.org/, and Epidemiology at http://www.epidem.com/). Information on the STROBE Initiative is available at www.strobe-statement.org.
